# Evaluating Nuclei Concentration in Amyloid Fibrillation Reactions Using Back-Calculation Approach

**DOI:** 10.1371/journal.pone.0020072

**Published:** 2011-05-20

**Authors:** Mirco Sorci, Whitney Silkworth, Timothy Gehan, Georges Belfort

**Affiliations:** Howard P. Isermann Department of Chemical and Biological Engineering and Center for Biotechnology and Interdisciplinary Studies, Rensselaer Polytechnic Institute, Troy, New York, United States of America; Consejo Superior de Investigaciones Cientificas, Spain

## Abstract

**Background:**

In spite of our extensive knowledge of the more than 20 proteins associated with different amyloid diseases, we do not know how amyloid toxicity occurs or how to block its action. Recent contradictory reports suggest that the fibrils and/or the oligomer precursors cause toxicity. An estimate of their temporal concentration may broaden understanding of the amyloid aggregation process.

**Methodology/Principal Findings:**

Assuming that conversion of folded protein to fibril is initiated by a nucleation event, we back-calculate the distribution of nuclei concentration. The temporal *in vitro* concentration of nuclei for the model hormone, recombinant human insulin, is estimated to be in the picomolar range. This is a conservative estimate since the back-calculation method is likely to overestimate the nuclei concentration because it does not take into consideration fibril fragmentation, which would lower the amount of nuclei

**Conclusions:**

Because of their propensity to form aggregates (non-ordered) and fibrils (ordered), this very low concentration could explain the difficulty in isolating and blocking oligomers or nuclei toxicity and the long onset time for amyloid diseases.

## Introduction

Even though the ancient Greek and Roman philosophers associated old age with increasing dementia, it was not until 1901 that German psychiatrist Alois Alzheimer connected amyloid fibrils with dementia [1]. Since then, considerable progress has been made in characterizing amyloid diseases [2]–[4]. Together with β-amyloid peptides [5], [6], many other proteins are part of the amyloid family: Islet amyloid polypeptide (type 2 diabetes mellitus) [7], α-synuclein (Parkinson's disease) [6], prions (transmissible spongiform encephalopathy) [8] and huntingtin (Huntington's disease) [9]. However, the connection between amyloidosis and disease is still unclear. Recently, oligomeric precursors of fibrillation have been proposed as possible toxic agents responsible for disease [10], [11]. Detailed analysis of these species, however, is still missing, mainly because of the inherent experimental challenge associated with isolation and structural characterization of individual components in a dynamic multi-component equilibrium. In this study, recombinant human insulin as a model amyloid protein was used, whose fibrillation mechanism is well characterized [12]–[15]. As early as 1957, Waugh proposed that a nearly simultaneous interaction of three to four insulin monomers forms a nucleus [16]. Later many other groups used different techniques to identify oligomeric species: Electrospray mass-spectroscopy (ES MS) [17], dynamic light scattering (DLS) [18], [19], atomic force microscopy (AFM) [20], synchrotron small angle X-ray scattering (SAXS) [21] and small angle neutron scattering (SANS) [22]. Among these, SAXS and SANS have advantages for obtaining structural information and allowed Vestergaard et al. and Nayak et al. to show the presence of oligomers as building blocks during insulin fibrillation [21], [22]. To overcome the different limitations of these techniques (e.g. the need for high sample concentration, the difficulty in characterizing mixtures, the requirement that samples must be stable during the time of the analysis and not be degraded by unwanted temperature increases), researchers have recently developed fluorescent methods [23] and used monoclonal antibodies [24] to capture the intermediate oligomeric species. However, no general consensus has been reached. Here, we offer a conservative theoretical estimate of nuclei concentrations using a “reverse calculation” in which fibril lengths are used to back-calculate the concentration of nuclei originally present during aggregation. The method is conservative because could overestimate the nuclei concentration, which would be lower (roughly 1 order of magnitude) when fibril breakage is considered as in some recent amyloid models [25]–[27]. No one, to our knowledge, has used this relatively simple method previously.

## Results

### Observations and assumptions

Previously we have studied insulin fibrillation, a process characterized by the following multiple stages: (i) a lag phase in which the nucleation process proceeds and no detectable fibrils are formed, (ii) an explosive elongation phase in which different lengths of fibrils are formed over a time period often shorter than the lag phase, and (iii) a saturation phase when elongation is terminated as most soluble protein is converted into fibrils [28]. Here, we question whether the end point of the process could provide information on the earlier stages, and specifically the nuclei concentration. We have observed the following: (i) Samples taken at different times along the oligomer reaction path (lag phase) exhibit *linearly* changing behaviour, supporting the idea of having in solution transient reactive species, which are the prerequisite for forming structurally rearranged intermediates and then fibrils; (ii) When aggregated species (oligomers/fibrils) reach a certain size they do not interact with each other within the time constraints of the measurement, probably because of significantly slower diffusion rates [28]. Instead, they react vigorously with free monomers, dimers or other small oligomers, in an elongation process that is many orders of magnitude faster than the nucleation process [29]. Defining the nucleus as the smallest oligomeric entity, on which fibril-like structures are built, we then assume that each fibril is generated by one nucleus and the number of nuclei corresponds to the number of the formed fibrils. Equivalent definitions of the nucleus are present in the literature: (i) the least thermodynamically stable species in solution, which is the oligomer of minimal size capable of initiating further growth [30] and (ii) the aggregate size after which the association rate exceeds the dissociation rate for the first time [31]. All these assumptions are independent of the particular size of the nucleus, which is also matter of debate since researchers have proposed critical sizes up to 40 monomers [32].

### Total number of nuclei

**Figure 1:**
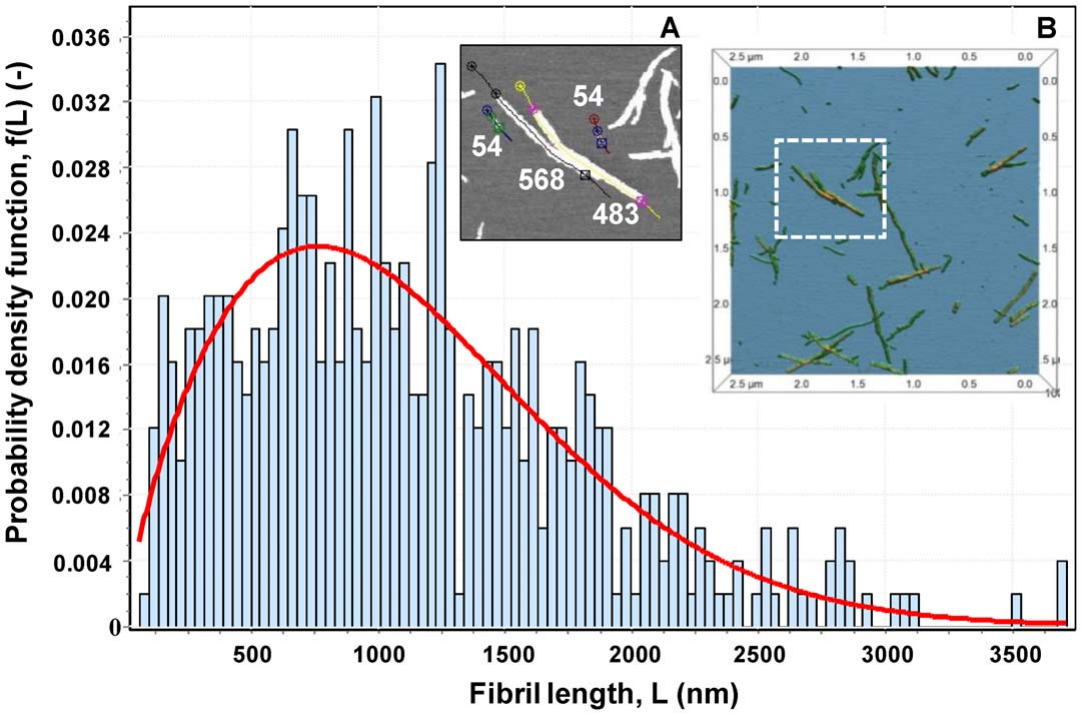
Fibril length distribution. The histogram of frequency versus fibril length summarizes AFM data for 495 insulin fibrils in 36.6 nm/bin for a total of 100 bins. The parameters of this distribution were estimated using distribution-fitting software, EasyFit (MathWave Technologies). The software fitted the data using 60 different distributions and ranked the results based on three different goodness-of-fit tests. The histogram shows the best fit (Kolmogorov-Smirnov statistic, *D* = 0.0187, Anderson-Darling, *A^2^* = 0.323, and Chi-Squared, *χ^2^* = 5.113) using the Weibull distribution (line). The probability density function is 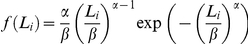 with values of the parameters: α = 1.7409 and β = 1248.5. (A) Example of a 2D AFM image of insulin fibrils, with measurements: A free-hand curve was drawn on the fibril and two cursors placed at each fibril end. Measurements are in nm. (B) Example of a 3D image, which assisted in detecting individual fibrils.

**Figure 2:**
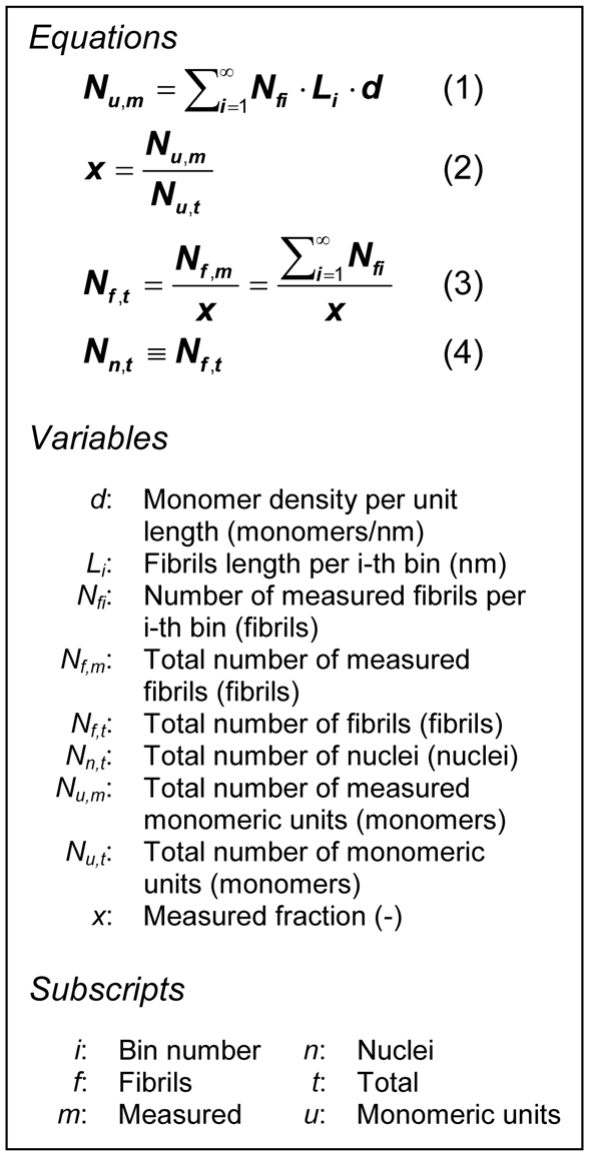
Equations and variables. Set of equations used to estimate the total number of insulin nuclei, *N_n,t_*, from the available fibril length distribution. The number of measured fibrils per i-th bin, *N_fi_*, Eqs. (1) & (3), were calculated using the Weibull distribution (**Figure 1**). From the definition of the nucleus, the total number of fibrils, *N_f,t_*, is equivalent to the total number of nuclei, *N_n,t_*, Eq. (4). A description and the units are provided for each variable.

The challenge then is to estimate the number and length of fibrils in solution. To do this, we first collected fibrils that were produced with our standard protocol for insulin fibrillation, using 2 mg/ml insulin solution, acidic buffer (pH 1.6) and high temperature (65±2°C). Samples were incubated for at least 5 hours, in order to reach the saturation phase. Then AFM was used to characterize the fibril length distribution. The data was fitted with 60 different distribution models [33], and the Weibull model with the best goodness-of-fit characteristics (i.e. Kolmogorov-Smirnov, Anderson-Darling and Chi-Squared) was chosen. Assuming the measured system (**Figure 1**) represents the whole sample, and knowing what fraction of the system has been measured, this allows us to estimate the total number of fibrils, and consequently the number of nuclei, assuming each fibril was generated by a single nucleus. Using electron microscopy, X-ray fibril diffraction, and biochemical studies, Ivanova et al. [34] proposed a model for fibrils of human insulin comprising two molecules per 4.7 Å layer of the fibril. This distance was suggested as one of the most conserved features of all amyloid fibril structures since 1968 [35]. This information can be converted into monomer density per unit fibril length, *d* = 2/0.47 = 4.26 monomer/nm. Using this information and the fibril length distribution (**Figure 1**), one can estimate *N_u,m_*, the number of monomeric units involved in the analyzed system. From Eq. (1) (**Figure 2**), *N_u,m_* = 2.33*10^6^ monomers, when considering data up to a cumulative function of 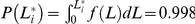 for L_i_^*^ = 3566 nm, where *f*(*L*) is the probability density function. This number of monomers is only a fraction, *x*, of all the monomers in the whole system. For an initial 2 mg/ml of insulin, or 0.000344 M (M.W. 5808 Da), the total number of monomers present in solution was *N_u,t_* = 0.00034*(6.023*10^23^)/1000 = 2.074*10^17^ monomers/ml. For 1 ml, from Eq. (2) (**Figure 2**) the fraction measured in the sample shown in **Figure 1**, *x* = 2.33*10^6^/2.074*10^17^ = 1.123*10^−11^. With this fraction of the whole sample, one can estimate the total number of fibrils, *N_f,t_*, present in the whole sample and therefore the number of nuclei, *N_n,t_*, from Eqs. (3) & (4) (**Figure 2**). From **Figure 1**, the total number of measured fibrils (up to 99.8% of the area under the curve) of different lengths in the sample is obtained from the Weibull distribution, *N_f,m_* = 492. Hence, the total number of fibrils formed in the whole sample was *N_f,t_* = *N_n,t_* = 492/*x* = 492/1.123*10^−11^ = 4.38*10^13^. In terms of concentration, we obtain the total nucleus concentration, *c_n,t_* = 4.38*10^13^/(6.023*10^23^)/0.001 = 7.27*10^−8^ M = 73 nM over the whole period.

### Time-dependent concentration of nuclei

**Figure 3:**
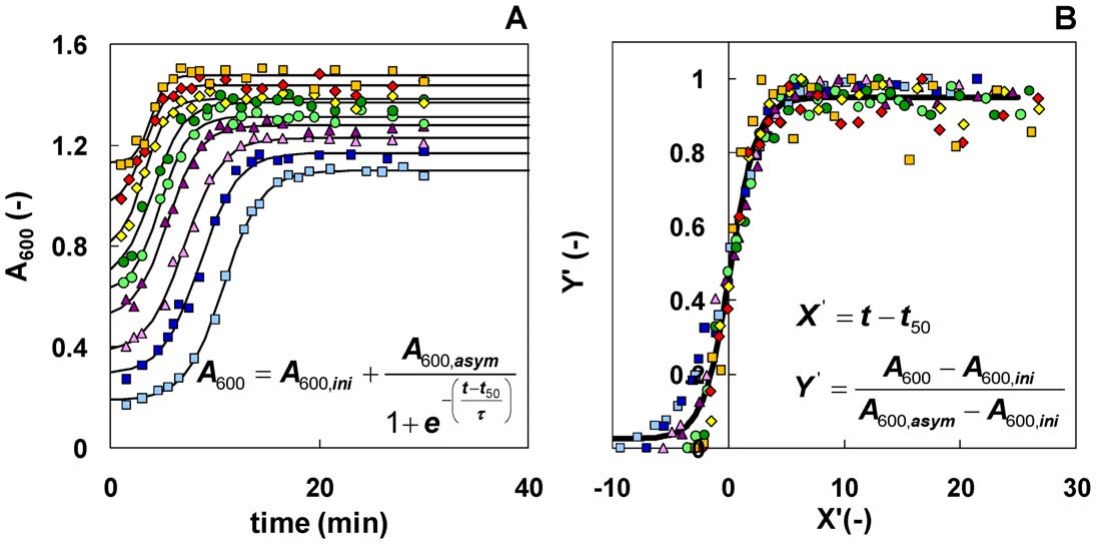
Elongation rate of fibrils with different length. A fresh 2 mg/ml insulin solution was seeded using fibrils with different length: 1L (light blue square), 1.5L (dark blue square), 2L (pink triangular), 2.5L (dark purple triangular), 3L (light green circle), 3.5L (dark green circle), 4L (yellow diamond), 4.5L (red diamond), 5L (orange square). The number of fibrils added as seeds was kept constant in all runs. (A) The elongation process was followed with absorbance at 600 nm (*A_600_*) and fitted with an empirical model (lines) already introduced in the literature [15], [28]. The equation is reported as an insert: *A_600,ini_* is the *A_600_* at the beginning of the experiment, *A_600,asym_* is the asymptotic *A_600_* at the end of the fibrillation process, *t* is the time, *t_50_* marks the middle of the fibrillation process and *τ* is representative of the sigmoid curve slope during the fibril growth phase. (B) After normalization of the data, using the new coordinates *X′* and *Y′* reported as an insert, all runs overlap and can be fitted with a universal sigmoidal curve derived from the same empirical model (line): Parallel slopes (*k_app_* = 1/*τ* = 0.86 min^−1^, with an *R^2^* = 0.87 from a linear fitting) is an indication of similar elongation rates, independent from the length of the fibrils used.

**Figure 4:**
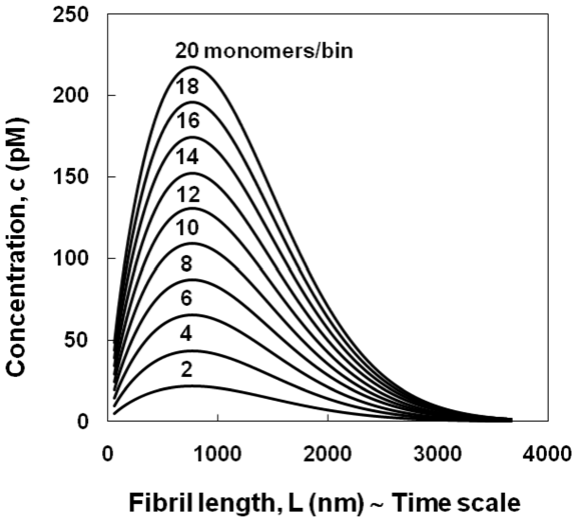
Nuclei concentration. Calculated profiles of nuclei concentrations (pM) versus length (nm), or equivalently time scale, as a function of the bin size. 2 monomers/bin corresponds to 0.47 nm/bin, while 20 monomers/bin corresponds to 4.7 nm/bin.

Why do fibrils have different lengths? To answer this question, we designed a set of seeding experiments, where we generated fibrils with different lengths and followed their elongation process. Since our results show that different length fibrils have similar elongation rates (**Figure 3**), it seems reasonable to hypothesize that fibril length is related to nucleus formation. For example, assuming that fibrils with a length of 1500 nm were generated by nuclei formed in solution at time t^*^, then (i) fibrils with *L*>1500 nm were generated by nuclei formed at *t*<t^*^, giving them the time to grow longer than 1500 nm; while (ii) fibrils with *L*<1500 nm were generated by nuclei formed at *t*>t^*^, i.e. these nuclei were not present in solution at t^*^. Hence, the fibrils length scale (**Figure 1**, horizontal-axis) is equivalent to a time scale. Reading the probability density function profile in **Figure 1** from right to left, the amount of nuclei at the beginning of the nucleation process was very small (long fibrils), it increased during the lag phase until it reached a maximum (at *L*∼900 nm), and finally it decreased (short fibrils), most likely because of the presence of mature fibrils formed during earlier stages for which the elongation process was much faster than the rate of nuclei formation. This trend is comparable to the results by Vestergaard et al. [21], who found a helical insulin oligomeric species accumulating and reaching a concentration maximum during the elongation phase. Our assumption suggests that all the nuclei calculated above, *N_n,t_*, are not present in solution at the same time, but represent an integral of all nuclei during the experiment. Hence, we refine our analysis (above), keeping in mind that the fibril count, *N_fi_* or vertical-axis (**Figure 1**), is related to nuclei concentration and the length scale, *L_i_* or horizontal-axis, to the time scale. The results, however, depend on the choice of a bin size, which determines the amount (nuclei concentration) of single elements, i.e. nuclei formed at different time points during the fibrillation process. In agreement with the model for fibrils of human insulin proposed by Ivanova et al. [34], with two molecules per 4.7 Å length of the fibril, the smallest bin size possible is the 2 monomers/bin, which corresponds to a 0.47 nm/bin. Using this bin size, we estimated a maximum concentration of 22 pM for the defined nuclei. Obviously, the choice of the bin size is arbitrary and ultimately depends on the elongation rate of fibrils. Since we have shown that elongation rates for the different lengths of fibrils are very similar (**Figure 3**), it is reasonable to assume 2 monomers/bin. However we have investigated the possibility that fibrils, even of the same length, had different elongation rates. As a consequence, nuclei present at the same time will generate fibrils with different lengths, and this difference would determine the correct bin size to choose. We have considered larger bin sizes, as multiples of the previous bin size, up to 20 monomers/bin (equivalent to 4.7 nm/bin and to a 116.16 kDa/bin), whose maximum concentration is 10 times larger than with 2 monomers/bin, but still in the pM range (**Figure 4**).

## Discussion

We have estimated a maximum concentration of insulin nuclei in the pM range. This result can be extended to oligomeric species larger or smaller than the nucleus. For the smaller oligomeric species that can self-interact and generate nuclei, their concentration has to be higher than that predicted here. However, their number (concentration) is likely to be the same order of magnitude as that of the nuclei. This is supported by recent results from our group, where oligomeric entities appear to be present below the 5 pM detection limit for the electrospray differential mobility analysis method (ES-DMA) [36]. Moreover, most of the protein maintains its original conformational structure and adds directly to the nuclei and fibrils in the elongation process, without passing through a series of intermediate to large oligomers. Hence, even though the formation of nuclei takes time (lag-phase) and there are very few nuclei in solution (pM range), the small native protein with its large value of diffusivity is quickly consumed as a simple building block. This is also confirmed by Sorci et al. [28].

### Supporting information and validation

Unfortunately data on time-dependent insulin nuclei concentration are not available to date, in order to validate our approach through a direct comparison between prediction and experimental data. Also experimental conditions and the definition of a nucleus change from author to author. Some considerations, however, can be still made. Nettleton and co-authors were able to identify the presence of oligomeric species using ES MS [17]. Before heating the sample, mass spectra showed predominantly monomeric and dimeric insulin with lower proportions of tetramer, pentamer and hexamer. After heating for 30 min at 70°C, the intensity of the hexamer and tetramer charge states were reduced relative to those of the pentamer. After 1.5 h, the presence of higher oligomers was significantly reduced and after 2 h monomeric insulin was the only species that could be observed in the mass spectrum. As the authors pointed out, the proportion of aggregating species detected by mass spectrometry was relatively low in comparison with monomeric species, with the intensity associated with the 12-mer generally being less than 0.1% of the signal for the monomer. These results cannot be used to validate our estimates here, but the lack of higher molecular weight oligomers support the idea that the concentration is probably below the detection limit of the instrument. As mentioned above, we obtained similar results using ES-DMA [36]. Using DLS both Grudzielanek et al. [18] and Ahmad et al. [19] showed that the oligomeric peak, which appears as monomeric/dimeric insulin, decreases and is quite broad, indicating the presence of a heterogeneous mixture of oligomers. However a distribution of the individual species is again not provided. Similar consideration is indicated from SAXS [21] and SANS [22] data. Podestá et al. calculated oligomers distributions for insulin aggregation combining time-resolved AFM and static light scattering [20], resulting in a steady-state distribution with an exponential tail until the formation of amyloid fibrils. The median aggregation size at different time points was 5.9, 4.9 and 6.7 nm, but the authors also pointed out that small species were filtered out by the edge-detection-algorithm used, which would decrease the medium aggregation size and the percentage of larger aggregates. Using a different technique, single molecule fluorescence, Orte et al. showed similar oligomer size distribution during aggregation of the SH3 domain of PI3 kinase [32]. The distribution of sizes for detected oligomers in solution was very broad and followed a log-normal function, peaking at an average size of 30 monomers. These data qualitatively support our approach, since our fibril distributions suggest a similar trend for the oligomeric species. On the other hand, they cannot be directly used to validate our estimates, since (i) they are distributions of oligomers of different sizes and not of nuclei (1 defined size) versus time, and (ii) they comprise frequency data without a mass balance to translate into concentration data as we have done.

### A conservative estimate

As in this work, the amyloid fibrillation process has been traditionally analyzed according to the classic nucleation-dependent polymerization model known as the “Oosawa's model” [37]. However, other and more complicated mechanisms have been proposed: Colloidal coagulation, downhill polymerization, and secondary nucleation (branching, fragmentation and heterogeneous nucleation) [31]. In particular, especially in the last decade, some researchers have proposed that the fibrillation kinetics is controlled in large part by the rate of fibril fragmentation, where fibrils break during the elongation phase and represent an additional source of new filaments in solution [25]–[27]. In this fragmentation model, multiple fibrils are generated by a single nucleus, while our more simplified theory proposes a one-to-one correspondence between fibrils and nuclei. According to our estimate, the time-dependent concentration of nuclei reaches a maximum value no larger than 250 pM and as small as 22 pM, or smaller if fibril breakage contributes significantly to the fibril formation mechanism. For example, consider the recent work on modeling insulin fibrillation by Knowles and co-authors, and the rate of multiplication of the filament population, 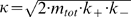, where *m_tot_* is the total monomer concentration, *m_tot_k_+_* the elongation rate and *k_−_* the fragmentation rate [27]. In that work, insulin fibrils were formed at 60°C and pH 2.0, close to our experimental conditions. Using the parameters *k_+_* = 2.9*10^4^ M^−1^ s^−1^ and *k_−_* = 2.1*10^−9^ s^−1^ (estimated by Knowles and co-authors) and *m_tot_* = 2/5808 = 3.44*10^−4^ M = 344 µM (since we used 2 mg/ml insulin in solution), one can estimate a rate of multiplication of the filament population *κ* = 0.000205 s^−1^. Thus, it takes 1/*κ* = 4883 s = 1.35 h to multiply the population of the filaments. Since our fibrillation process is completed in about 5–6 hours, the fibril population can multiply 4 times at most and 1 nucleus will generate 2^4^ = 16 fibrils, decreasing our original estimate by about 1 order of magnitude only. In other words, our picomolar concentration predictions for the nuclei are conservative compared with more complicated models that account for fragmentation. Also, all secondary mechanisms are strongly dependent on stirring and fibrils present in the solution, neither of which was involved in generating the fibrils used in our calculation: Samples were not agitated during fibrillation and seeds were not added to the fresh insulin solution at the beginning of the experiment.

### Limitations

We suggest that the reason nuclei are still structurally uncharacterized, is not only because they belong to a very dynamic multi-component system, but also because of low experimental resolution. This analysis is based on the nucleation model, the structural design of which is unknown [21], [22]. We also do not know how such nuclei convert to fibrils, which at least for insulin do not appear to mostly grow symmetrically from each end [38]. Clearly circular structures like those proposed by Quist et al. [39] may have difficulty elongating, while asymmetric structures may elongate more easily [38]. Recently, Meng et al. [40] have demonstrated that 1.0 µm diameter hard spheres with short-range attraction in water formed asymmetric clusters rather than symmetric ones. For 6 spheres (two groups independently suggest that nuclei comprise 6 monomers or 3 dimers) [21], [22], they observed two final structures both with 12 contacts and similar potential energy; one asymmetric (poly-tetrahedron) structure and one symmetric (octahedron) structure at 96% and 4% cluster probability, respectively. It seems that the asymmetric structures are likely to form fibrils than the symmetric ones (see the Vestergaard et al. [21] paper and supplementary movie demonstration fibril growth with asymmetric hexamer nuclei). The approach presented here is not exhaustive and there is room for refinement (e.g. knowing precise elongation rates of fibrils and the exact bin size for the statistical analysis, including the contribution of the secondary fragmentation mechanism in estimating the total amount of nuclei). The results do not answer questions like “which is the correct nucleation mechanism?” or “which entity (the oligomer or the fibril) is the toxic one?”, but aim to help researches to better target these oligomeric entities, and point out the need for new analytical techniques to investigate them. In this direction Lindgren and Hammarström describe fluorescent probes as a new tool for sensitive detection in real-time of oligomeric precursor [23]. This would also provide experimental data to check the validity of our approach and estimates.

### Conclusion

A conservative theoretical “reverse” calculation to estimate nuclei concentration, during the lag-phase of insulin aggregation, is proposed based on experimental results (AFM fibrils distribution and seeding experiments to investigate elongation rates of fibrils with different lengths) and a structural fibril model proposed in the literature [34]. For insulin nuclei, defined here as the precursor to the initiation of fibril formation, they are present in the pM concentration range. Even lower concentration would occur when fibril breakage is considered. The approach is general and could be extended to other amyloid systems. Our calculation is the first attempt, to our knowledge, to quantify the nuclei concentration and may broaden understanding of amyloids aggregation.

## Materials and Methods

### Reagents

Human recombinant insulin was provided by Novo Nordisk A/S, Denmark. NaCl and HCl were certified ACS reagent grade (Fisher Scientific, Pittsburgh, PA and Sigma-Aldrich, St. Louis, MO, respectively). Buffer solutions were filtered prior to use through a 0.22 µm poly(ether sulfone) membrane filter (Millipore Corp., Bedford, MA).

### The standard protocol for insulin fibrillation

Each kinetic experiment was performed at 65±2°C with fresh 2 mg/ml insulin solution in 100 mM NaCl and 25 mM HCl (pH 1.6), prepared immediately prior to use. Fibrils production was followed by monitoring the increase in suspended matter via absorbance at 600 nm (*A_600_*). As a complementary measurement, fibrils were removed by centrifugation (12,000 rpm, 15 min) and the supernatants were assayed at *A_280_* to measure insulin depletion. Complete description of the protocol and of the resulting sigmoidal curve is provided in previous work [28]. The fibrils used here were collected from insulin samples incubated at 65±2°C for at least 5 hours, to assure the asymptotic phase was reached, and without centrifugation, to avoid breakage of the fibrils. Absorbance readings were performed using a UV–vis spectrophotometer (Hitachi U-2000, Hitachi High Technologies America, Inc., San Jose, CA).

### Seeding experiment

This set of experiments was designed to generate, in a controlled way, different length fibrils and then study their elongation mechanism. The protocol comprises three steps. *Step 1* – Generating fibrils: In order to generate fibrils with different length, we first collected “1L-fibrils” at the end of a standard kinetic experiment. These fibrils are also known in terms of mass (e.g. 2 mg in a 1 ml solution) and are characterized by their length at the maximum frequency. *Step 2* – Elongating fibrils: Longer fibrils were then generated adding amounts of fresh insulin to a known amount of “1L-fibrils” (e.g. for “2L-fibrils”: 2 mg of “1L-fibrils” +2 mg of fresh insulin) and allowing the fibrils to elongate. The assumption is that the system did not form a significant amount of new nuclei, but just elongated the pre-existing “1L-fibrils”. This assumption is supported by the different time-scales of the two phenomena: It takes hours to form nuclei and then fibrils, while the elongation process is completed in minutes. *Step 3* – Seeding fibrils: Finally fibrils with different length were added as seeds to a fresh 2 mg/ml insulin solution. The number of fibrils added as seeds was kept constant in all runs. The elongation process was followed with absorbance at 600 nm (*A_600_*).

### Atomic force microscopy

Images of insulin fibrils (inserts) were obtained with an AFM (MFP-3D, Asylum Research, Santa Barbara, CA) and standard Si cantilevers (AC240TS, Olympus America Inc., Center Valley, PA). Samples were diluted 1∶100 with deionized water and then an aliquot of 20 µL was placed on a mica surface for adsorption for 5 min. Non adsorbed protein was washed away with deionized water. Three dimensional measurements were collected in air using the tapping mode technique of AFM. 2D images were analyzed with Igor Pro software (Wavemetrics Inc., Portland, OR) for estimates of fibril length.
